# Cognitive and Affective Changes in Mild to Moderate Alzheimer’s Disease Patients Undergoing Switch of Cholinesterase Inhibitors: A 6-Month Observational Study

**DOI:** 10.1371/journal.pone.0089216

**Published:** 2014-02-19

**Authors:** Gianfranco Spalletta, Carlo Caltagirone, Alessandro Padovani, Sandro Sorbi, Mahmood Attar, Delia Colombo, Luca Cravello, on behalf of the E V O L U T I O N study Working Group

**Affiliations:** 1 Neuropsychiatry Laboratory, Department of Clinical and Behavioral Neurology, IRCCS Santa Lucia Foundation, Rome, Italy; 2 Department of System Medicine, University of Rome “Tor Vergata”, Rome, Italy; 3 Department of Clinical and Experimental Sciences, University of Brescia, Brescia, Italy; 4 Department of Neurological and Psychiatric Sciences, University of Florence, Florence, Italy; 5 Novartis Farma Italia, Origgio (Varese), Italy; “Mario Negri” Institute for Pharmacological Research, Italy

## Abstract

Patients with Alzheimer’s disease after an initial response to cholinesterase inhibitors may complain a later lack of efficacy. This, in association with incident neuropsychiatric symptoms, may worsen patient quality of life. Thus, the switch to another cholinesterase inhibitor could represent a valid therapeutic strategy. The aim of this study was to investigate the effectiveness of the switch from one to another cholinesterase inhibitor on cognitive and affective symptoms in mild to moderate Alzheimer disease patients. Four hundred twenty-three subjects were included from the EVOLUTION study, an observational, longitudinal, multicentre study conducted on Alzheimer disease patients who switched to different cholinesterase inhibitor due either to lack/loss of efficacy or response, reduced tolerability or poor compliance. All patients underwent cognitive and neuropsychiatric assessments, carried out before the switch (baseline), and at 3 and 6-month follow-up. A significant effect of the different switch types was found on Mini-Mental State Examination score during time, with best effectiveness on mild Alzheimer’s disease patients switching from oral cholinesterase inhibitors to rivastigmine patch. Depressive symptoms, when measured using continuous Neuropsychiatric Inventory values, decreased significantly, while apathy symptoms remained stable over the 6 months after the switch. However, frequency of both depression and apathy, when measured categorically using Neuropsychiatric Inventory cut-off scores, did not change significantly during time. In mild to moderate Alzheimer disease patients with loss of efficacy and tolerability during cholinesterase inhibitor treatment, the switch to another cholinesterase inhibitor may represent an important option for slowing cognitive deterioration. The evidence of apathy stabilization and the positive tendency of depressive symptom improvement should definitively be confirmed in double-blind controlled studies.

## Introduction

Alzheimer’s disease (AD) is a severe chronic neurodegenerative disease characterized by progressive cognitive impairment, functional decline and neuropsychiatric symptoms [1].

The phenomenology of AD is greatly variable due to the influence of many factors, such as comorbid non-degenerative medical diseases, concomitant pharmacological treatments, environmental variables and progression of dementia itself [2]. All these factors, in association with treatment response, determine a high heterogeneity of clinical manifestations and very often make it difficult to manage patients [3]. Thus, the effectiveness of AD treatment, especially with regard to neuropsychiatric phenomenology, is of fundamental importance not only to reduce patient suffering and caregiver burden but also to contain economic costs of the disease [4].

Cholinesterase inhibitors (ChEIs), the most effective drugs available at present for treatment of mild to moderate AD, can stabilize cognitive symptoms for a one to three year period, but they are not able to modify the progression of the disease [5]. There is also preliminary evidence that they may improve some neuropsychiatric symptoms [6]. Unfortunately, the therapeutic response to ChEIs is less satisfying in the long-term period and some patients adhere to prescribed treatment for only a short time. Poly-pharmacotherapy due to comorbid diseases, side effects particularly caused by high dosages of oral ChEIs, and initial or delayed lack of efficacy are some reasons of reduced compliance [7].

One of the possible strategies to improve compliance and effectiveness in patients no longer responding to initial treatment is the switch from one to another ChEI [7]. To date, few studies have focused on ChEI switch and the vast majority of results have concentrated on cognitive impairment and described a switch from donepezil to rivastigmine, suggesting that patients non responder to donepezil may benefit from the switch [8], [9]. In particular, the switch from oral to transdermal formulation was demonstrated to be effective on cognitive symptoms and to have a good safety profile [10].

Furthermore, little is known on the effects of a switch on neuropsychiatric symptoms. In particular, no data are available on the effectiveness on the two more common symptoms in AD, which is depression and apathy. Thus, we collected data from the “bEhaVioral symptOms in Alzheimer’s disease: evaLUation of paTIents treated with chOliNesterase inhibitors” (EVOLUTION) study in order to describe changes in cognitive and affective domain severity in mild to moderate AD patients enrolled in a switch ChEI study for lack or loss of efficacy and tolerability/compliance.

## Materials and Methods

### Ethics Statement

The study was approved by the ethical committees of the three coordinating centres, that is: IRCCS Fondazione Santa Lucia, Roma, Università of Firenze, and Università di Brescia, and by the ethical committees of all the participating memory clinics of the EVOLUTION study group, that is: Ospedale Garibaldi, Catania, Ospedale Civile Guzzardi, Ragusa, Ospedale Mazzini, Teramo, Ospedale dell’Annunziata, L’Aquila, Ospedale Civile San Pio da Pietralcina, Chieti, AORN Cardarelli, Napoli, Policlinico Universitario Tor Vergata, Roma, AORN S.Sebastiano, Caserta, Ospedale San Salvatore, L’Aquila, Università degli Studi di Torino Clinica Neurologica, Ospedale San Filippo Neri, Roma, ASL RM F, Roma, Ospedale S. Giovanni Calibita, FBF, Roma, Ospedale Sant’Anna, Como, Ospedale Niguarda Ca’ Granda, Milano, Ospedale Santa Scolastica, Frosinone, Ospedali Riuniti, Bergamo, Ospedale Santa Corona, Savona, Policlinico Martino, Messina, Ospedale San Pietro FBF, Roma, Policlinico Consorziale, Bari, Azienda Ospedaliera di Verona, Ospedale Civile Agnelli, Torino, Ospedale Evangelico Valdese, Torino, Distretto 2, Modena, Ospedali Riuniti, Ancona, Ospedale Galliera Mura delle Cappuccine, Genova, Policlinico SS Annunziata, Chieti, Azienda Ospedaliera Padova, AUOP Policlinico Giaccone, Palermo, Ospedale degli Infermi, Rimini, Ospedale Manzoni, Lecco, Ospedale Centrale di Bolzano, Ospedale Morgagni Pierantoni, Forlì Cesena. All included subjects and/or caregivers signed an informed consent form prior to enrolment, in accordance with the Helsinki Declaration.

The EVOLUTION observational study meets the ethical-administrative Italian legislation at the time of the study administrative process start (03.11.2009) according to “CM 6 02.09.2002, GU 214 12.09.2002” and “D 29.03.2008” of the Agenzia Italiana del Farmaco (AIFA – Italian Medicines Agency) GU 76 31.03.2008, Art 10 (Procedures for Observational Studies).

### Methods

The EVOLUTION study is an observational, longitudinal, multicentre study conducted in 38 outpatient memory clinics throughout Italy. To be eligible in the study the subjects met the following inclusion criteria: 1) diagnosis of probable AD according to the National Institute of Neurological and Communicative Disorders and Stroke and the Alzheimer’s Disease and Related Disorders Association (NINCDS-ADRDA) criteria [11]; 2) mild to moderate severity of dementia, defined as Mini-Mental State Examination (MMSE) [12] score ranging from 26 to 10; 3) onset of symptoms occurred at least 6 months before the date of the enrolment; 4) patient treated with ChEI for at least 6 months, performing for the first time a switch to another ChEI due to lack of response (i.e., lack or loss of efficacy defined as a reduction of at least 2 points of MMSE score in the last 6 months) [13] and/or reduced compliance (due to side effects or no adherence to recommended oral dosing regimen); 5) vision and hearing sufficient for compliance with testing procedures; 6) presence of caregiver able to understand all testing procedures. Exclusion criteria were: 1) hospitalization (i.e., to be an inpatient); 2) history of head trauma or other neurologic diseases apart from AD; 3) clinically significant or unstable major medical illnesses (e.g., diabetes, obstructive pulmonary disease or asthma, hematologic disorders, active gastrointestinal, renal, hepatic, endocrine or cardiovascular disorders); 4) history of cancer within the last 5 years; 5) dementia other than probable AD; 6) known or suspected history of alcoholism or drug dependence and abuse during lifetime.

All included patients underwent cognitive and neuropsychiatric assessments, carried out before the switch (baseline), and at 3 and 6-month follow-up. Trained psychologists and neuropsychologists performed all evaluations.

The global cognitive impairment was assessed by the MMSE [12], a widely used neurocognitive screening test measuring orientation, language, verbal memory, attention, visuospatial function and mental control. MMSE score ranges in 11 different items and lower scores mean higher cognitive impairment. Based on MMSE total score the patients were classified as having mild (MMSE = 18–26) or moderate (MMSE = 10–17) AD.

The Neuropsychiatric Inventory (NPI) was used to assess the frequency and severity of neuropsychiatric symptoms in 12 domains: delusions, hallucinations, agitation, depression, anxiety, euphoria, apathy, disinhibition, irritability, aberrant motor behavior, nighttime behavior disturbances, appetite and eating abnormalities [14]. The severity and frequency of each symptom were scored on the basis of structured questions administered to the caregiver. Frequency was rated from 1 (occasionally) to 4 (very frequently) and severity from 1 (mild) to 3 (severe). If the symptom was absent, a score equal to zero was given. The multiplication of frequency and severity was used as symptom composite score, with a range from 0 to 12. We used two different methods to investigate neuropsychiatric phenomena. First, we measured continuous scores of NPI. Second, NPI symptoms were also categorized (Yes/No) on the basis of criteria useful for clinical purposes in AD patients [15]. In particular, symptom composite score ≥4 indicates the presence of clinically relevant symptoms, typically associated with therapeutic intervention, a score between 1 and 3 characterizes mild symptoms usually not requiring specific treatment, and a score of 0 means no symptoms [16], [17].

For this study, two different switch types were considered: 1) from non-rivastigmine oral ChEI (i.e. donepezil and galantamine) to rivastigmine transdermal patch, and 2) from rivastigmine patch to other non-rivastigmine oral ChEI. According to the treatment guidelines for AD, the practice was that patients switched immediately from other ChEI to rivastigmine transdermal patch 4.6 mg/24 h; one month after the switch, they were given a dose increase to 9.5 mg/24 h and remained on this dosage, unless they experienced adverse events. Patients who experienced adverse events had rivastigmine patch dosage reduced to 4.6 mg/24 h. About the second switch type, only data on patients switching from rivastigmine transdermal patch to donepezil were considered: the practice was that patients switched from rivastigmine transdermal formulation to 5 mg/24 h oral donepezil following a 7 days withdrawal period. After one month donepezil was titrated to 10 mg/die and maintained at this dosage through the study, unless adverse events appeared. Patients who experienced adverse events had donepezil dosage reduced to 5 mg/24 h.

Differences among variables at baseline were measured by means of chi-square for categorical variables and Analysis of Variance (ANOVA) with Bonferroni post-hoc test (significance corrected for multiple comparisons) for continuous variables. For the aims of this study we focused our neuropsychiatry analyses only on the two more common symptoms in AD, that is depression and apathy, as measured by NPI. Thus, a series of 3 repeated measures ANOVAs with MMSE, NPI depression/dysphoria or NPI apathy scores as dependent variables and categories of AD severity (mild/moderate) and switch type (oral/patch) as independent variables were used to assess cognitive and affective (apathy-depression) changes during time between groups with different switches. The level of statistical significance was defined as p<0.05.

## Results

### Patients’ Characteristics, Comorbid Illnesses, and Concomitant Treatments

Of the overall group of the 635 subjects enrolled in the EVOLUTION study (mean age 77±7 SD years; 60% women), 423 patients satisfied the inclusion/exclusion criteria of the present sub-analysis and were here considered (mean age 78±5 SD years; 60% women).

Based on the type of switch and on the severity of AD, four groups of patients were identified: 1) switch from oral ChEI to rivastigmine patch with mild AD; 2) switch from oral ChEI to rivastigmine patch with moderate AD; 3) switch from rivastigmine patch to oral ChEI with mild AD; and 4) switch from rivastigmine patch to oral ChEI with moderate AD.

Sociodemographic features of the four groups are summarized in Table 1.

**Table 1:** Sociodemographic and clinical characteristics of the four switch subtypes.

	Switch from oral to patch ChEI		Switch from patch to oral ChEI	
	Mild AD	Moderate AD	Mild AD	Moderate AD
	(n = 165)	(n = 201)	(n = 22)	(n = 35)
	Mean ± SE	Mean ± SE	Mean ± SE	Mean ± SE
Age (years)	77.2±0.4	78.7±0.4	78.2±1.4	79.5±1
Female n° (%)	85 (51.5)	134 (66.7)	12 (54.6)	25 (71.4)
Education n° (%)				
none	16 (9.7)	37 (18.4)	2 (9.1)	5 (14.3)
primary education	84 (50.9)	119 (59.2)	12 (54.6)	22 (62.9)
lower secondary education	32 (19.4)	23 (11.4)	4 (18.2)	2 (5.7)
upper secondary education	24 (14.6)	15 (7.5)	2 (9.1)	4 (11.4)
tertiary education	9 (5.5)	7 (3.5)	2 (9.1)	2 (5.7)
MMSE score	21±0.2	14.3±0.2	20.1±0.4	13.9±0.4
NPI depression/dysphoria score	1.7±0.2	2.2±0.2	1.5±0.5	1.7±0.5
NPI apathy score	3.4±0.3	4.0±0.3	3.7±0.8	4.5±0.6

AD = Alzheimer’s disease; ChEI = cholinesterase inhibitor; MMSE = Mini-Mental State Examination; NPI = Neuropsychiatric Inventory; SE = standard error.

Reasons of the switch therapy were: loss of ChEI efficacy (n = 175, 41.4%), lack of response (n = 122, 28.8%), reduced tolerability (n = 60, 14.2%), and poor compliance (n = 42, 9.9%). In 24 (5.7%) patients other causes determined the switch (i.e. patient/caregiver request, no dose regimen reached in previous treatment). Thus, in more than 70% of the included AD patients causes of cognitive decline during the last 6 months before the switch were lack of efficacy or response.

Comorbidity for other medical illnesses was present in about 75% of the whole patient group, and no significant differences emerged among the four subgroups. The most frequent comorbid illnesses were hypertension (n = 209, 49.4%), dyslipidemia (n = 67, 15.8%) and not complicated diabetes (n = 64, 15.1%); other concomitant stable illnesses, such as gastrointestinal, renal, pulmonary and metabolic diseases, accounted for less than 5% of patients.

One hundred eighty six (44%) patients reached the maximum dosage of ChEI (10 mg for oral donepezil, 9.5 mg for rivastigmine patch), with no significant difference among the four groups. Megadose regimen was not used in any patient. Patients switching to oral ChEI, with moderate AD, had significant longer duration of maximum dosage (147.8 days ±35.7 SD) than those switching to patch ChEI, both with mild AD (104.8 days ±39.6 SD; F = 7.278; df = 3; p<0.0001) and with moderate AD (111.3 days ±39.1 SD; F = 7.278; df = 3; p = 0.002), while no significant difference was found with patients switching to oral ChEI with mild AD (138.6 days ±42.4 SD). Only 11 patients (3%) belonging to both groups switching to patch ChEI reported clinically significant side effects, mainly immediately after the dosage increase: cutaneous rush (8 patients), malaise (1 patient), irritability (1 patient) and nausea (1 patient). In 6 patients the clinician made a temporary drug interruption with restart at lower dosages, in 2 patients a lower dosage was prescribed without washout period, in 3 patients no action was taken.

At the baseline visit, 36 (8.5%) patients of the whole patient group were treated with meantime and there was a significant difference among the four groups: patients switching to oral ChEI had higher frequency (mild AD: n = 3, 13.6%; moderate AD: n = 7, 20%) than those switching to patch ChEI (mild AD: n = 7, 4.2%; moderate AD: n = 19, 9.5%) (Chi-Square = 10.766; df = 3; p = 0.013). The mean daily dosage of memantine was 15.9 mg ±5.1 SD). The only changes in memantine treatment during the 6-month observational period were made, in a total of 10 (2.4%) subjects, at the 3-month follow-up visit. In particular, 5 patients (2 switching to patch ChEI with mild AD, 1 switching to patch ChEI with moderate AD, and 2 switching to oral ChEI with moderate AD) had a new prescription of memantine, while 5 patients (2 switching to patch ChEI with mild AD, 3 switching to patch ChEI with moderate AD, and 1 switching to oral ChEI with mild AD) withdrawn memantine due to side effects. Thus, these few changes in memantine prescription during the six-month period of observation may be considered irrelevant for the results of this study.

At the baseline visit, 126 patients (29.8%) were under psychotropic medications with no statistically significant differences among the four groups (Chi-Square = 2.844; df = 3; p = 0.416). The psychotropic agents more frequently used were antidepressants (n = 88, 20.8%) and antipsychotics (n = 49, 11.6%). benzodiazepines or hypnotics were prescribed in 17 patients (4%).

### Cognitive and Neuropsychiatric Symptoms

At baseline no significant differences were found among four groups of patients identified according to switch modality (from oral to patch ChEI, from patch to oral ChEI) and dementia severity (mild AD, moderate AD) except for MMSE scores: as expected, patients with moderate AD had significant lower MMSE scores than those with mild AD (F = 389.184; df = 1,419; p<0.0001). Moreover, patients switching from oral to patch ChEI had slightly higher MMSE scores than patients switching from patch to oral ChEI (F = 3.910; df = 1,419; p<0.0486) (see Table 1).

**Figure 1:**
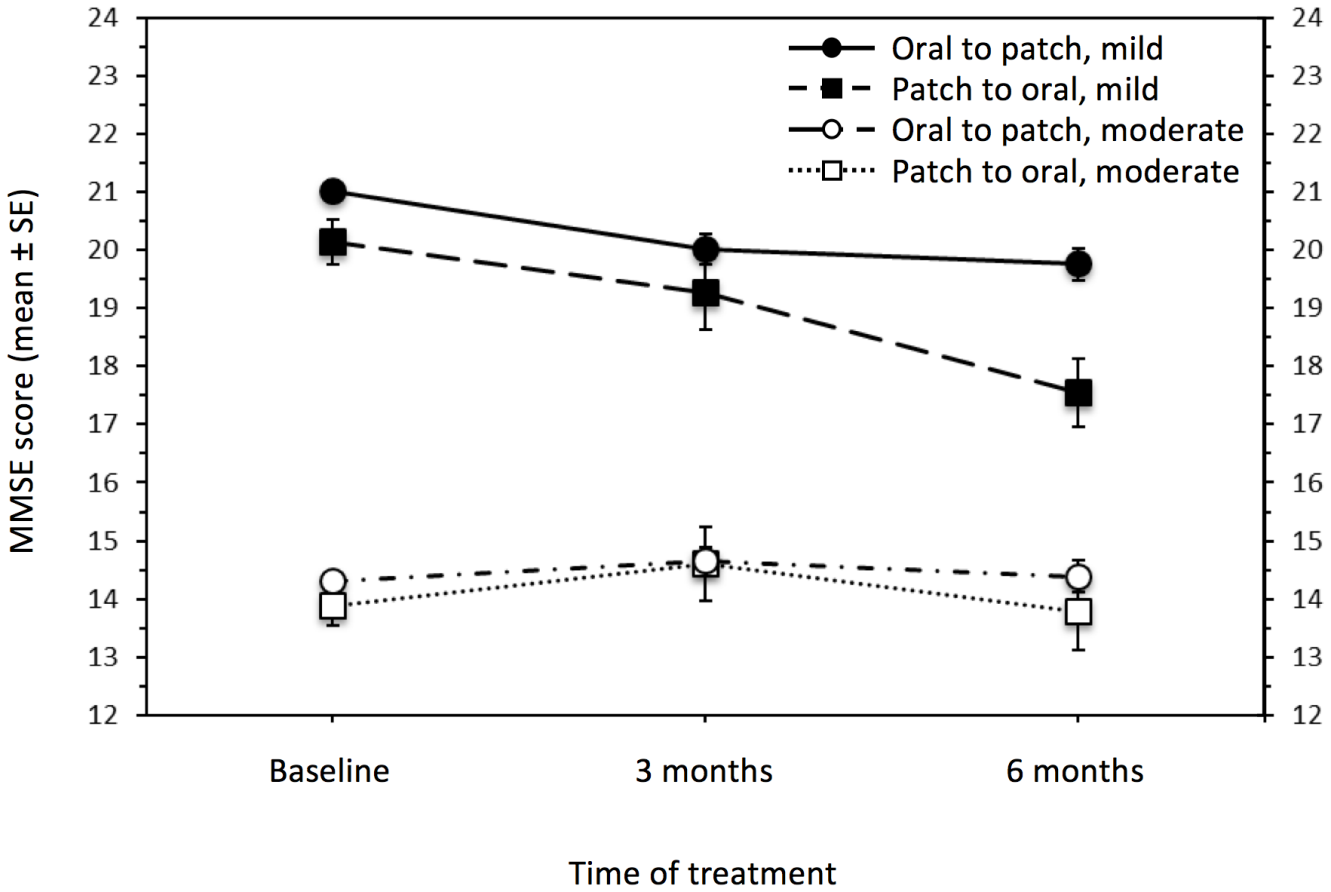
Changes of global cognitive level during ChEI switch. An analysis of variance with repeated measures indicates that the longitudinal course of global cognitive level (i.e. MMSE score changes during a 6-month period) in mild to moderate AD patients performing for the first time a switch to another ChEI due to lack of response/reduced compliance, is more favourable when switching from oral ChEIs to rivastigmine patch, with best effectiveness in mild AD patients (see the results section for statistic details). SE = Standard Error; MMSE = Mini-Mental State Examination.

Mild AD patients switching from rivastigmine patch to non-rivastigmine oral ChEI (n = 22) had a mean MMSE score of 20.1 (±0.4 SE) at the baseline, decreasing to 17.5 (±0.6 SE) at 6-month follow-up (delta = 2.6) in comparison of mild AD patients with opposite switch (n = 165) who decreased from 21.0 (±0.2 SE) to 19.8 (±0.3 SE) (delta = 1.2). MMSE score changes in patients with moderate AD were overlapped between groups with different switch modalities (see Figure 1).

There was a significant change in MMSE score during time (F = 11.4; df = 2,838; p<0.0001; lambda = 22.795; power = 0.997), a significant interaction between MMSE score change and categories of AD severity (F = 3.0; df = 2,838; p = 0.049; lambda = 6.067; power = 0.579), and a significant interaction between MMSE score change and groups of different switches (F = 10.7; df = 2,838; p<0.0001; lambda = 21.338; power = 0.995).

**Figure 2:**
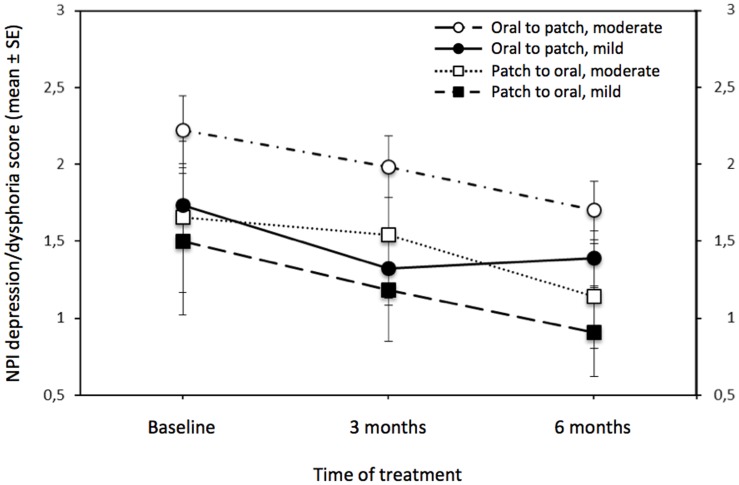
Changes of NPI depression/dysphoria score during ChEI switch. An analysis of variance with repeated measures indicates that the longitudinal course of depressive symptoms (i.e. NPI depression score changes during a 6-momth period) in mild to moderate AD patients performing for the first time a switch to another ChEI due to lack of response/reduced compliance, improves independently from AD severity and switch type (see the results section for statistic details). SE = Standard Error; NPI = Neuropsychiatric Inventory.

NPI depression/dysphoria continuous scores decreased significantly during time (F = 4.0; df = 2,838; p = 0.019; lambda = 8.004; power = 0.717) from 1.9 (±0.1 SE) to 1.4 (±0.1 SE), with no interaction effect (see Figure 2).

**Figure 3:**
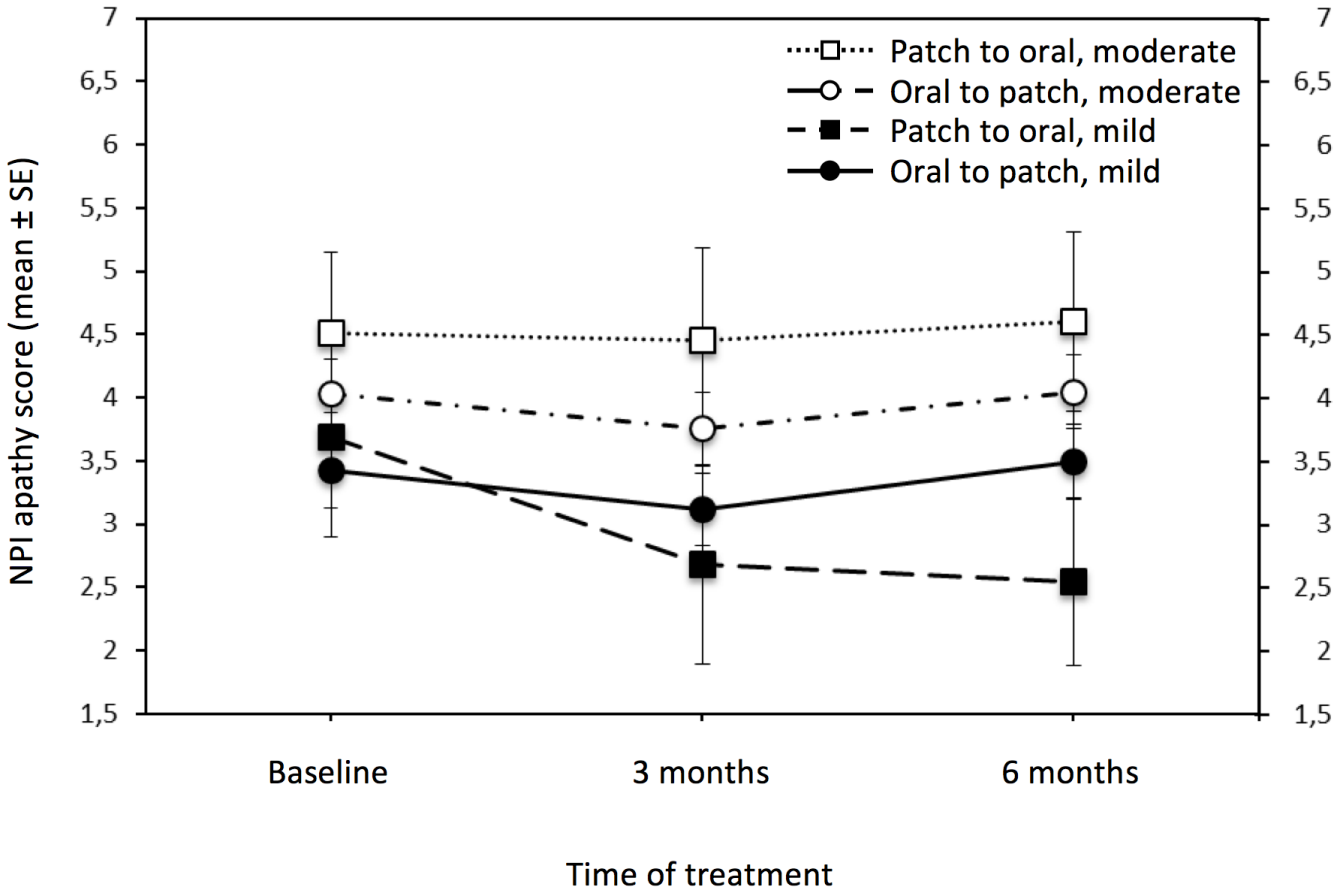
Changes of NPI apathy score during ChEI switch. An analysis of variance with repeated measures indicates that the longitudinal course of apathy symptoms (i.e. NPI apathy score changes during a 6-momth period) in mild to moderate AD patients performing for the first time a switch to another ChEI due to lack of response/reduced compliance, do not change over time (see the results section for statistic details). SE = Standard Error; NPI = Neuropsychiatric Inventory.

NPI apathy continuous scores were very stable during time (F = 1.6; df = 2,838; p = 0.2; lambda = 3.218; power = 0.328) with no variation in score from 3.8 (±0.2 SE) to 3.8 (±0.2 SE), with no interaction effect (see Figure 3).

Detailed frequencies of categories of depression and apathy at the baseline and follow-up are described in Table 2. In particular, according to NPI categorization, clinically relevant symptoms of depression (score≥4) were present in 111 patients (26.2%) at baseline and in 84 patients (19.9%) at 6-month follow-up. In addition, apathy clinically relevant symptoms (score≥4) were present in 207 patients (48.9%) at baseline and in 200 patients (47.3%) at 6-month follow-up. Differences in frequency from baseline to 6-month follow-up for each category of depression and apathy did not reach statistical significance either in the subgroups of mild and moderate AD or in the total groups (p>0.1 for all comparisons) (see Table 2).

**Table 2:** Frequency of depression and apathy, as categorized using Neuropsychiatric Inventory ≥4 cut-off scores, in 423 patients with AD undergoing switch ChEI therapy.

Switch	AD severity	Depression						Apathy					
		Baseline			6 month follow-up			Baseline			6 month follow-up		
		Nosymptoms	Mildsymptoms	Clinicallyrelevantsymptoms	Nosymptoms	Mildsymptoms	Clinicallyrelevantsymptoms	Nosymptoms	Mildsymptoms	Clinicallyrelevantsymptoms	Nosymptoms	Mildsymptoms	Clinicallyrelevantsymptoms
		n (%)	n (%)	n (%)	n (%)	n (%)	n (%)	n (%)	n (%)	n (%)	n (%)	n (%)	n (%)
Switch fromoral to patchChEI (n = 366)	Mild AD	95 (57.6)	31 (18.8)	39 (23.6)	98 (59.4)	36 (21.8)	31 (18.8)	67 (40.6)	31 (18.8)	67 (40.6)	65 (39.4)	24 (14.5)	76 (46.1)
	Moderate AD	106 (52.7)	34 (16.9)	61 (30.3)	119 (59.2)	35 (17.4)	47 (23.4)	72 (35.8)	21 (10.4)	108 (53.7)	72 (35.8)	32 (15.9)	97 (48.3)
Subtotal		201 (54.9)	65 (17.8)	100 (27.3)	217 (59.3)	71 (19.4)	78 (21.3)	139 (38.0)	52 (14.2)	175 (47.8)	137 (37.4)	56 (15.3)	173 (47.3)
Switch frompatch to oralChEI (n = 57)	Mild AD	10 (45.5)	9 (40.9)	3 (13.6)	13 (59.1)	7 (31.8)	2 (9.1)	7 (31.8)	4 (18.2)	11 (50)	10 (45.5)	4 (18.2)	8 (36.4)
	Moderate AD	23 (65.7)	4 (11.4)	8 (22.9)	21 (60)	10 (28.6)	4 (11.4)	6 (17.1)	8 (22.9)	21 (60)	9 (25.7)	7 (20)	19 (54.3)
Subtotal		33 (57.9)	13 (22.8)	11 (19.3)	34 (59.6)	17 (29.8)	6 (10.5)	13 (22.8)	12 (21.1)	32 (56.1)	19 (33.3)	11 (19.3)	27 (47.4)
Total		234 (55.3)	78 (18.4)	111 (26.2)	251 (59.3)	88 (20.8)	84 (19.9)	152 (35.9)	64 (15.1)	207 (48.9)	156 (36.9)	67 (15.8)	200 (47.3)

No symptoms: NPI = 0; Mild symptoms: NPI = 1–3; Clinically relevant symptoms: NPI≥4. AD = Alzheimer’s disease; ChEI = cholinesterase inhibitor; NPI = Neuropsychiatric Inventory.

Chi-square analyses indicated no statistical significance differences between baseline and 6-month follow-up (p>0.1 for all comparisons).

## Discussion

Results of this observational study suggest that in AD patients no more responsive to initial treatment, with reduced tolerability and/or compliance and loss of at least 2 points at MMSE score in the last 6 months, the switch from oral ChEI (donepezil or galantamine) to transdermal rivastigmine patch formulation may reduce the progression of global cognitive impairment, particularly in mild AD patients. In addition, stabilization in the frequency of clinically relevant depression and apathy phenomena may be achieved by using switch procedure, independently from the switch type and the illness severity. There is also a preliminary suggestion that improvement in continuous values of depressive symptoms may be achieved, but this point needs of further validation.

Several studies have been conducted in order to monitor and describe the utilization of ChEIs and their switch in the clinical practice. A recent retrospective study, carried out on a US administrative database of 3177 AD patients treated at least once with ChEI, showed that the switch from one ChEI to another one ranges from 14.5% to 21.5%, with a mean time of treatment with the same drug ranging between 226 and 206 days for the three different ChEIs (donepezil, rivastigmine and galantamine) [18]. Previous studies, always on administrative databases, showed that approximately 30% of patients treated with donepezil or rivastigmine drop out treatment or perform a switch to another ChEI within 60 days after the start of the first therapy with ChEI [19]. Some observational studies highlighted that the switch from other ChEIs to rivastigmine leads to evident cognitive benefits and, in some cases, to tolerability improvement of the new treatment [13], [20]. More recently, the switch from oral to transdermal ChEI formulation has been demonstrated to be effective and with a good safety profile [10]. Tian et al, in a retrospective study performed on 772 AD patients initially treated with donepezil, showed that the switch to rivastigmine transdermal patch resulted in a greater adherence to the treatment regimen compared to oral treatment [21]. This result was more evident in patients who carried the switch in the first year after they started therapy with oral donepezil [21]. Moreover, the switch from a ChEI in oral formulation to rivastigmine transdermal patch can be made immediately without washout period [22].

The results of our study enforce all these previous data on the favourable effect of the switch from oral ChEI to rivastigmine transdermal patch. Moreover, the advantage of ChEI switch is more evident if we consider that in the last 6 months before baseline visit almost 70% of patients had a reduction of at least 2 points of MMSE score, while after the switch a stabilization of cognitive functions was present. Indeed, we found that patients with AD (particularly of mild severity) who switched from non-rivastigmine oral ChEI to rivastigmine transdermal formulation had lower progression of cognitive impairment than those switching from transdermal to oral formulation. One of the possible explanation of the positive effect of the switch from non-rivastigmine oral to rivastigmine transdermal ChEI is that transdermal formulation allows a continuous release of the active compound, thus avoiding fluctuations in plasma levels that are typical of the oral formulation, moreover it limits the typical side effects of oral ChEIs and increases patient compliance [10]. The rivastigmine effectiveness on patients no more responsive to other ChEI could be explained also from a molecular point of view. Indeed, rivastigmine has pharmacological properties that distinguish it from other ChEI. First, rivastigmine has a selective effect on G1 enzymatic isoform of acetylcholinesterase, that predominates in patients with AD [23]; second, rivastigmine inhibits not only acetylcholinesterase but also butyrylcholinesterase that is able of compensating for acetylcholinesterase function in case of deficiency [24]; third, the low protein binding property of rivastigmine is indicative of reduced drug interactions [23] and higher compliance, especially in elderly AD patients who typically have concomitant illnesses and take multiple medications [25].

Finally, the cognitive effect of rivastigmine was strongly evident on patients with mild illness severity at the baseline, possibly because of the rivastigmine effect on butyrylcholinesterase. We can speculate on mechanisms under this effect. Indeed, in two dated papers has been hypothesized that butyrylcholinesterase might play a role in the aggregation of beta-amyloid that occurs especially in the early stages of AD [26], [27]. In addition, results of two recent preclinical studies more clearly indicate possible mechanisms of beneficial effects of rivastigmine treatment, describing: a) protection from change of neuronal morphology and presynaptic protein markers in degenerating primary embryonic cerebrocortical cultures [28], and b) enhancement of neuronal secreted Abeta Precursor Protein (APP), wich is protective against neuronal apoptosis, and shift APP processing toward the a-secretase pathway, both phenomena which mirror the trend of synaptic proteins, and metabolic activity [29]. Further, on the basis of preliminary data on structural brain changes in AD patients treated with ChEI [30], we could also speculate that treatment with dual ChEI, such as rivastigmine, may decrease the rate of brain atrophy by the reduction of amyloid plaque neurotoxicity due to butyrylcholinesterase inhibition. This effect, if present, should be more evident in patients at the early stage of the disease.

Another interesting result highlighted from the EVOLUTION study is the improvement of depressive symptoms (limited to the analysis of continuous values of NPI scores) and the stabilization of apathy symptoms within 6 months after switch procedures. This effect was observed independently from the switch type and the illness severity. Some pieces of evidence in the literature suggest that depression in AD has peculiar features and it is closely linked to the primary neurodegenerative disease. In fact, post-mortem studies demonstrated that patients with AD and history of major depression show an increased number of neurofibrillary tangles and beta-amyloid plaques, the characteristic neuropathological markers of AD, in hippocampus [31]. Furthermore, in patients with major depression at the time of AD clinical diagnosis, the presence of such markers is further increased [31]. These data clearly explain the negative results of recent studies focused on therapeutic efficacy of antidepressants in patients with AD [32], [33] and, on the other hand, could justify an important role of the drugs currently used for the treatment of cognitive impairment on the control of depressive symptoms in patients with AD. In this regard, a recent study evaluated the effect of rivastigmine transdermal formulation in patients with AD and comorbid major depressive episode (MDE), never treated before with antidepressants [34]. After six months of treatment with rivastigmine transdermal patch a significant reduction of the occurrence of MDE and a reduction of the severity of depressive symptoms were evident. In addition, this pattern was more evident in patients largely responsive to treatment with rivastigmine, indicating a possible positive effect of the drug on AD patients with depressive phenomenology [34].

As far to apathetic symptoms, no clinical studies on the possible positive effect of ChEI switch have yet been conducted. Moreover, the association between apathy and dementia has been largely demonstrated in recent studies [35], [36], so as some authors postulated that apathy should be considered a mixed cognitive/psychiatric disturbance related to AD neurodegeneration [37]. Thus, apathetic symptoms, as well as cognitive symptoms, could benefit from switch to a more effective ChEI.

Some limitations of the present study deserve to be mentioned. First, this is an observational study with the well-known limitations derived from this type of study. In particular, we did not include a control group of patients with no switch from one ChEI to another. Thus, further double blind controlled studies, including patients steadily treated with antidementia drugs, should confirm results here reported. Second, the sample of patients who switched from oral to transdermal ChEI was much greater than those performing the opposite switch. Although this difference may potentially influence our results, it is possible that clinicians used more frequently the oral to transdermal ChEI switch because their feelings of a better effectiveness and/or less occurrence of side effects of transdermal formulation compared to the oral one, and this is in line with the final results. Third, we missed a control group of patients without switch or free from anti-dementia treatment (i.e. for side effects, loss of efficacy or no compliance). Strength of the study was the design, comprehensive of cognitive and psychiatric assessments at 6-month follow-up examinations.

In conclusion, results of this arm of the EVOLUTION study suggests that, in mild to moderate AD patients with lack or loss of efficacy and/or tolerability to one ChEI treatment, the switch to another ChEI may represent an important tool in the therapeutic treatment of cognitive and even affective symptoms. This possibility should be better evaluated in further controlled studies.
